# Neogastropod phylogenetic relationships based on entire mitochondrial genomes

**DOI:** 10.1186/1471-2148-9-210

**Published:** 2009-08-23

**Authors:** Regina L Cunha, Cristina Grande, Rafael Zardoya

**Affiliations:** 1 Departamento de Biodiversidad y Biología Evolutiva, Museo Nacional de Ciencias Naturales-CSIC, José Gutiérrez Abascal, 2, 28006 Madrid, Spain; 2 CCMAR, Campus de Gambelas-Universidade do Algarve, 8005-139 Faro, Portugal; 3 Centro de Biología Molecular Severo Ochoa; Nicolás Cabrera, 1, Universidad Autónoma de Madrid; 28049 Madrid, Spain

## Abstract

**Background:**

The Neogastropoda is a highly diversified group of predatory marine snails (Gastropoda: Caenogastropoda). Traditionally, its monophyly has been widely accepted based on several morphological synapomorphies mostly related with the digestive system. However, recent molecular phylogenetic studies challenged the monophyly of Neogastropoda due to the inclusion of representatives of other caenogastropod lineages (e.g. Littorinimorpha) within the group. Neogastropoda has been classified into up to six superfamilies including Buccinoidea, Muricoidea, Olivoidea, Pseudolivoidea, Conoidea, and Cancellarioidea. Phylogenetic relationships among neogastropod superfamilies remain unresolved.

**Results:**

The complete mitochondrial (mt) genomes of seven Neogastropoda (*Bolinus brandaris*, *Cancellaria cancellata*, *Conus borgesi*, *Cymbium olla*, *Fusiturris similis*, *Nassarius reticulatus*, and *Terebra dimidiata*) and of the tonnoidean *Cymatium parthenopeum *(Littorinimorpha), a putative sister group to Neogastropoda, were sequenced. In addition, the partial sequence of the mitochondrial genome of the calyptraeoidean *Calyptraea chinensis *(Littorinimorpha) was also determined. All sequenced neogastropod mt genomes shared a highly conserved gene order with only two instances of *tRNA *gene translocation. Phylogenetic relationships of Neogastropoda were inferred based on the 13 mt protein coding genes (both at the amino acid and nucleotide level) of all available caenogastropod mitochondrial genomes. Maximum likelihood (ML) and Bayesian inference (BI) phylogenetic analyses failed to recover the monophyly of Neogastropoda due to the inclusion of the tonnoidean *Cymatium parthenopeum *within the group. At the superfamily level, all phylogenetic analyses questioned the taxonomic validity of Muricoidea, whereas the monophyly of Conoidea was supported by most phylogenetic analyses, albeit weakly. All analyzed families were recovered as monophyletic except Turridae due to the inclusion of Terebridae. Further phylogenetic analyses based on either a four mt gene data set including two additional Littorinimorpha or combining mt and nuclear sequence data also rejected the monophyly of Neogastropoda but rendered rather unresolved topologies. The phylogenetic performance of each mt gene was evaluated under ML. The total number of resolved internal branches of the reference (whole-mt genome) topology was not recovered in any of the individual gene phylogenetic analysis. The *cox2 *gene recovered the highest number of congruent internal branches with the reference topology, whereas the combined *tRNA *genes, *cox1*, and *atp8 *showed the lowest phylogenetic performance.

**Conclusion:**

Phylogenetic analyses based on complete mt genome data resolved a higher number of internal branches of the caenogastropod tree than individual mt genes. All performed phylogenetic analyses agreed in rejecting the monophyly of the Neogastropoda due to the inclusion of Littorinimorpha lineages within the group. This result challenges morphological evidence, and prompts for further re-evaluation of neogastropod morphological synapomorphies. The important increase in number of analyzed positions with respect to previous studies was not enough to achieve conclusive results regarding phylogenetic relationships within Neogastropoda. In this regard, sequencing of complete mtDNAs from all closely related caenogastropod lineages is needed. Nevertheless, the rapid radiation at the origin of Neogastropoda may not allow full resolution of this phylogeny based only on mt data, and in parallel more nuclear sequence data will also need to be incorporated into the phylogenetic analyses.

## Background

Neogastropoda [1,2], also known as Stenoglossa [3], comprises a highly diverse group of predatory marine shelled gastropods with more than 16,000 living species [4] including e.g., cone snails (Conidae), balers (Volutidae), purple dye murex snails (Muricidae), augers (Terebridae), and whelks (Buccinidae) [5]. Neogastropoda are dominant in many benthic environments, and attain their maximum diversity in the tropical seas [6,7]. The most prominent feature of Neogastropoda is their active predatory behavior (most species are carnivorous), which was achieved after important morphological changes including e.g., the elongation of the siphonal canal, a shift in the mouth opening to a terminal position on the head, and the formation of a well-developed proboscis [5,8-10].

Ever since Thiele [1], Neogastropoda have been considered a natural group, clearly differentiated from other Caenogastropoda. The monophyly of the group is widely accepted among morphologists [5], and it is based on several synapomorphies mostly related with the anatomy of the digestive system [5,8,10-13]. Current classifications of Neogastropoda generally recognize up to six superfamilies: Buccinoidea, Muricoidea, Olivoidea, Pseudolivoidea, Conoidea, and Cancellarioidea [1,2,8,12,14] (Additional file 1). Phylogenetic relationships among neogastropod superfamilies based on morphological characters are rather unstable, and for instance, Cancellarioidea [8] or Buccinoidea [5] have been alternatively proposed as the sister group of the remaining Neogastropoda.

Thus far, molecular phylogenetic analyses based on relatively short fragments of both nuclear and mitochondrial (mt) DNA recovered rather unresolved topologies, which contradicted morphological evidence, and failed to support the monophyly of Neogastropoda [15-20]. For instance, the most complete molecular phylogenetic study thus far performed on this subject [21] was based on a multigene data set that included both nuclear (*18S rRNA*, *28S rRNA*, *EF1-α*, and *Histone H3*; 2,707 bp) and mt (*cox1 *and *12S rRNA*; 1,288 bp) sequences of 29 Caenogastropoda. The reconstructed tree failed to recover the monophyly of Neogastropoda, and two Littorinimorpha lineages (Tonnoidea and Calyptraeoidea) were recovered as the closest sister groups of two Neogastropoda lineages (Volutidae and Cancellariidae, respectively), although with low statistical support. In contrast, more recently, a phylogenetic analysis combining both morphological, and molecular data (using the same genes as in [21]) recovered Neogastropoda as monophyletic [5] stimulating the debate.

The available fossil record of Neogastropoda is quite thorough, and supports a widely accepted evolutionary scenario of an Early Cretaceous origin of the group followed by two rapid diversification rounds in the late Cretaceous and the Paleocene, respectively [22-24]. The successive bursts of cladogenesis in the evolutionary history of the group could be hampering successful recovery of phylogenetic relationships within the group. Given the relatively short internodes connecting main superfamilies of Neogastropoda and related caenogastropod lineages, it seems worthwhile gathering and analyzing larger sequence data sets in order to obtain more phylogenetic informative sites for trying to resolve the question at hand.

Phylogenetic analyses based on complete mt sequence data have proved to enhance resolution, and statistical confidence of inferred phylogenetic trees in vertebrates when compared with analyses based only on partial mt genes [25-27]. Within gastropods, mitogenomic data have demonstrated to be useful in recovering phylogenetic relationships within opisthobranchs (sea slugs), and in demonstrating the non-monophyly of pulmonates (snails) [28]. Moreover, gastropod mitochondrial genomes show a wide variety of gene arrangements, which, if shared-derived, could be used to infer phylogenetic relationships [5,28]. Hence, sequencing and analyzing mt genome data seems to be a promising tool for further addressing the controversy on neogastropod monophyly and phylogenetic relationships. Nevertheless, it is important to note that phylogenetic analyses based on mt genomes show limitations (particularly when addressing deep phylogenetic relationships but not only), and can render odd results that are incongruent with other evidences [29-31]. Unorthodox phylogenetic relationships based on mt genome data can be explained as artefacts due to e.g. incorrect rooting [32] or low signal-to-noise ratio [33], but are difficult to correct. Hence, it is widely accepted that phylogenetic results derived from mt genome data need to be confirmed with evidence based on nuclear genes, which have a slower evolutionary rate, and may show in some cases better phylogenetic performance (e.g. [34]). Thus far, however, only few nuclear markers such as e.g. *18S rRNA*, *28S rRNA*, *EF1-α*, and *Histone H3 *have been developed for phylogenetic studies in gastropods with limited success [15,16,20,21].

To date, only five entire mt genomes of Neogastropoda have been determined: *Conus textile *NC_009797, [35]; *Lophiotoma cerithiformis *NC_008098, [36]; *Ilyanassa obsoleta *NC_007781, [37], *Thais clavigera *NC_010090[38] and *Rapana venosa *NC_011193[39]. These mt genomes represent only two (Conoidea and Buccinoidea) out of the six currently recognized superfamilies of Neogastropoda. Moreover, no mt genomes are available for any of the putative caenogastropod sister group lineages of Neogastropoda.

In this study, we sequenced the entire mt genome of another seven neogastropod species that represent four out of the six currently recognized superfamilies (Muricoidea, Buccinoidea, Cancellarioidea, and Conoidea), as well as a representative of Tonnoidea (Littorinimorpha), one of the proposed sister groups to the Neogastropoda [11,40-42]. In addition, we sequenced a fragment of approximately 8,500 base pairs of the mt genome of a representative of Calyptraeoidea (Littorinimorpha), another potentially closely related taxon of Neogastropoda [21]. The new sequence data were used (both at the amino acid and nucleotide level) to reconstruct the phylogeny of Neogastropoda, and test its monophyly. The phylogenetic performance of individual mt genes was also investigated, and compared with that of whole mt genomes. In addition, evolution of mitochondrial gene arrangements within Gastropoda was revisited in the light of the new mt genomic data.

## Results

### Mitochondrial genome organization

**Figure 1:**
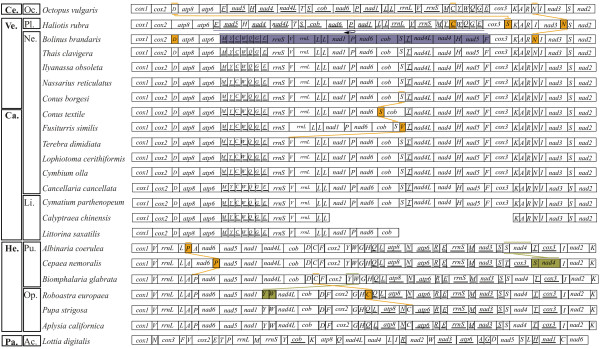
**Hypothesized gene rearrangements of gastropod mt genomes**. Genes encoded by the minor strand are underlined. Genes are colored to facilitate following main inversion and transposition events between Cephalopoda, Vetigastropoda, and Caenogastropoda. The many gene rearrangements that potentially occurred at the origin of Patellogastropoda and Heterobranchia, or between both groups and Caenogastropoda, are not shown. **Ce**.: Cephalopoda; **Ve**.: Vetigastropoda; **Ca**.: Caenogastropoda; **He**.: Heterobranchia; **Pa**.: Patellogastropoda; **Oc**.: Octopoda; **Pl**.: Pleurotomarioidea; **Ne**.: Neogastropoda; **Li**.: Littorinimorpha; **Pu**.: Pulmonata; **Op**.: Opisthobranchia; **Ac**.: Acmaeoidea.

The complete mt genomes of seven neogastropods (*Bolinus brandaris*, *Cancellaria cancellata*, *Conus borgesi*, *Cymbium olla*, *Fusiturris similis*, *Nassarius reticulatus*, and *Terebra dimidiata*) and of *Cymatium parthenopeum *(Littorinimorpha:Tonnoidea) were sequenced. The total lengths of the new sequences ranged between 15,270 and 16,648 bp (Table 1). All newly sequenced complete mt genomes encoded for 13 protein-coding, 22 transfer RNA (tRNA), and two ribosomal RNA (rRNA) genes. All neogastropod mt genomes shared the same gene order with only two exceptions (Fig. 1): (1) the *trnS (ucn) *gene from *Fusiturris similis *was positioned between *nad6 *and *cob *genes, whereas in the other neogastropod mt genomes is found between *cob *and *trnT*; (2) the position of the *trnV *in *Terebra dimidiata *was located between *trnS (ucn) *and *trnT*, whereas in the other neogastropod mt genomes is found between both rRNA genes. The mt genome of the tonnoidean *Cymatium parthenopeum *presented the neogastropod mt genome consensus gene order (Fig. 1). The partial sequence of the mt genome of *Calyptraea chinensis *(Littorinimorpha: Calyptraeoidea) was 8,530 bp in length, and comprised 25 out of the 37 mt genes. The deduced gene order of this partial mt genome was consistent with the neogastropod mt genome consensus gene order (from *trnK *to *trnL (uur)*) (Fig. 1). Moreover, the gene order of the partial sequence of *Littorina saxatilis *(Littorinomorpha: Littorinoidea) mt genome, which is available in GenBank (AJ_132137, [43], is also consistent with the neogastropod mt genome consensus gene order (from *cox1 *to *cob*) (Fig. 1).

**Table 1:** Main structural features of the eight mitochondrial genomes sequenced in the study

	*Bolinus brandaris*	*Cancellaria cancellata*	*Cymbium olla*	*Fusiturris similis*	*Nassarius reticulatus*	*Terebra dimidiata*	*Conus borgesi*	*Cymatium parthenopeum*
total size (bp)	15, 380	16, 648	15, 375	15, 595	15, 27	1 16, 510	15, 536	15, 270
%A	0.29	0.28	0.30	0.29	0.30	0.26	0.29	0.31
%C	0.15	0.12	0.14	0.16	0.16	0.13	0.15	0.15
%G	0.18	0.18	0.17	0.18	0.17	0.22	0.18	0.16
%T	0.38	0.42	0.39	0.37	0.38	0.40	0.38	0.38
%A+T	0.67	0.70	0.69	0.66	0.68	0.60	0.67	0.69
Potential origin of replication	60	112	120	51	57	848	127	15
Size range of gene overlapping	1 to 12	1 to 3	1 to 3	1 to 3	1 to 3	1 to 2	3	1 to 4

For each, total size of the mitochondrial genome, overall base composition, % of A-T-rich sequences, size of the potential origin of replication, and size range of gene overlapping are presented. Sizes are expressed as bp.

All genes of the newly sequenced complete mt genomes used ATG as start codon except *nad4*, which used other start codons (ATA in *Bolinus brandaris*, *Nassarius reticulatus*, and *Cymbium olla*, ATT in *Cancellaria cancellata *and *Conus borgesi*, and TTG in *Fusiturris simili*s, *Cymatium parthenopeum*, and *Terebra dimidiata*). In these mt genomes, only eigth out of 296 genes ended with incomplete stop codons (notably *nad4 *gene in *Fusiturris similis*, *Nassarius reticulatus*, and *Terebra dimidiata*). Two noncoding regions were found in all analyzed mitochondrial genomes between *trnF *and *cox3 *(14 – 848 bp in length), and between *atp6 *and *trnM *(27 – 44 bp in length). Another two noncoding regions were found in all analyzed mt genomes except *Cancellaria cancellata *between *cox1 *and *cox2 *(15 – 132 bp in length), and between *cox3 *and *trnK *(12 – 83 bp in length). In addition, a large noncoding region located between *nad1 *and *trnP *(869 bp in length) was found in *Cancellaria cancellata*. The potential origin of replication was located between *trnF *and *cox3 *(15 – 848 bp in length) by comparison with other gastropod mt genomes. Instances of overlapping between adjacent genes occurred in all newly sequenced mt genomes (Table 1). The *cox2 *and *trnD *genes overlap in every genome except that of *Calyptraea chinensis *whereas *trnW *and *trnQ *overlap in every genome except that of *Cymbium olla*. Overall base compositions of the newly sequenced mt genomes are shown in Table 1.

### Phylogenetic relationships of Neogastropoda based on complete mt genome sequences

Phylogenetic relationships among gastropod main lineages were inferred based on complete mt genome data both at the amino acid (13-protein data set) and nucleotide (allnuc data set) level using *Lottia digitalis *(Patellogastropoda), and *Haliotis rubra *(Vetigastropoda) as outgroups, respectively. Both data sets were analyzed under maximum likelihood (ML) and Bayesian inference (BI).

**Figure 2:**
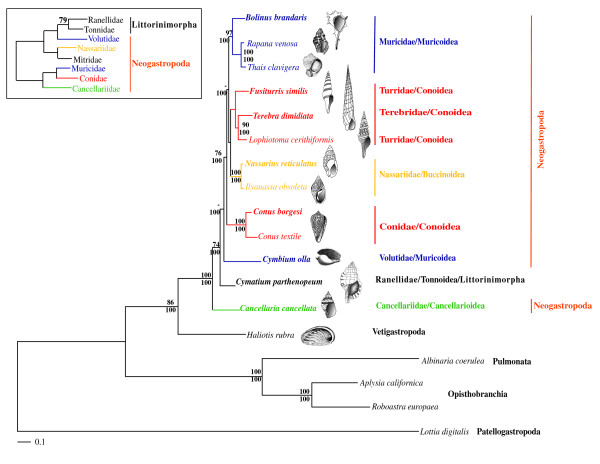
**Phylogenetic relationships within Gastropoda**. ML phylogram inferred from a single concatenated data set of deduced amino acid sequences of all 13 mitochondrial protein-coding genes (13-protein data set). Species whose complete mt genome was sequenced in this study are presented in bold. *Lottia digitalis *(Patellogastropoda) was used as outgroup. Numbers in the nodes correspond to ML bootstrap proportions (above branches) and BI posterior probabilities (below branches). Only values above 70% are represented. The inset shows a ML topology based on fragments of mt and nuclear data (adapted from Fig. two of Colgan *et al*., 2007).

Both, the ML (-LnL = 46918.42) and the BI (-LnL = 47950.14) trees that were reconstructed based on the 13-protein data set failed to recover the monophyly of Neogastropoda (Fig. 2) due to the inclusion of *Cymatium parthenopeum *(Littorinimorpha: Tonnoidea) within the group with strong support (74% bootstrap support (BP), and 100% Bayesian posterior probability (BPP), respectively). The monophyly of the superfamily Conoidea was only recovered in the BI analysis (with maximal support; not shown). Both, ML and BI analyses failed to recover the monophyly of the superfamily Muricoidea (Fig. 2). The status of other neogastropod superfamilies could not be tested in the present study since only one family represented them. Internal branches connecting neogastropod families were rather short, and phylogenetic relationships among superfamilies were rather unresolved. Only the relative most basal position of Cancellarioidea with respect to other superfamilies was strongly supported by both, ML and BI analyses (Fig. 2). At a lower taxonomic level, the monophyly of all analyzed neogastropod families was strongly supported by the 13-protein data set with the exception of Turridae, which was strongly rejected (90% BP and 100% BPP in the ML and BI analyses, respectively) due to the inclusion of Terebridae (Fig. 2).

**Figure 3:**
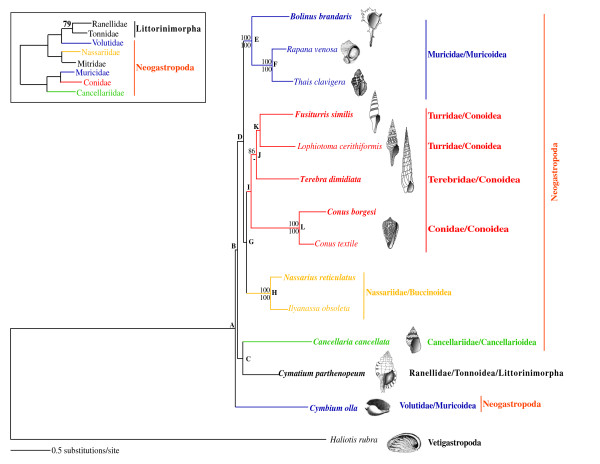
**Phylogenetic relationships within Gastropoda**. ML phylogram based on the nucleotide sequences of the rRNA, tRNA and protein-coding genes of all available complete mt genomes of neogastropods, and one Vetigastropoda (*Haliotis rubra*), chosen as outgroup (allnuc data set). Species whose complete mt genome was sequenced in this study are presented in bold. Numbers in the nodes correspond to ML bootstrap proportions (above branches) and BI posterior probabilities (below branches). Only values above 70% are depicted. The inset shows a ML topology based on fragments of mt and nuclear data (adapted from Fig. Two of Colgan *et al*., 2007). Letters A-K label nodes used in the analysis of phylogenetic performance.

Phylogenetic relationships within Caenogastropoda were also analyzed based on complete mt genome nucleotide sequence data (allnuc data set). Neogastropoda were not recovered as a monophyletic group due to the inclusion of the tonnoidean *Cymatium parthenopeum*, which was placed as sister group of Cancellariidae in both, ML (-lnL = 125810.87) and BI (-lnL = 124904.38) analyses (Fig. 3). However, this result was only strongly supported by BI when third codon positions of all protein coding genes were removed from the analysis (not shown). Both, ML and BI analyses based on the allnuc data set recovered Connoidea as a monophyletic group but without strong statistical support. In contrast, both ML and BI analyses rejected the monophyly of Muricoidea because Volutidae failed to group together with Muricidae (Fig. 3). This result only received strong bootstrap support when third codon positions of all protein coding genes were removed from the analysis (not shown). Phylogenetic relationships among superfamilies were largely unresolved in the reconstructed tree based on the allnuc data set (Fig. 3).

### Phylogenetic relationships of Neogastropoda based on partial mt genome sequences

**Figure 4:**
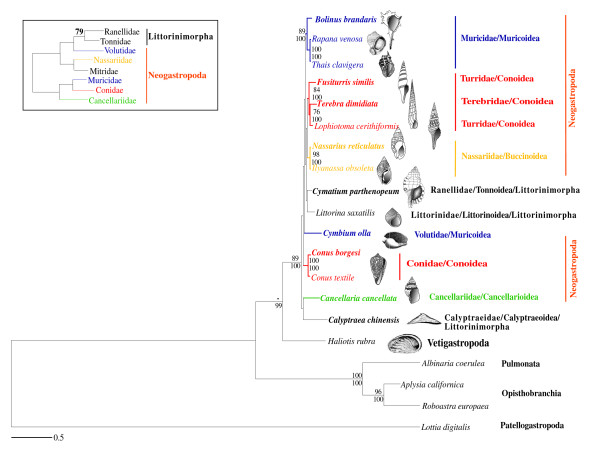
**Phylogenetic relationships within Gastropoda**. ML phylogram inferred from a single concatenated data set of deduced amino acid sequences of 4 mitochondrial protein-coding genes (4-protein data set). *Lottia digitalis *(Patellogastropoda) was used as outgroup. Numbers in the nodes correspond to ML bootstrap proportions (above branches) and BI posterior probabilities (below branches). Only values above 70% are represented. The inset shows a ML topology based on fragments of mt and nuclear data (adapted from Fig. Two of Colgan *et al*., 2007).

To further test the monophyly of Neogastropoda, we analyzed a 4-protein data set that included two extra Littorinimorpha species (*Calyptraea chinensis *and *Littorina saxatilis*), using *Lottia digitalis *as outgroup (see Material and Methods). Both, the ML (-Ln L = 12497.47) and BI (-Ln L = 12694.60) reconstructed trees could not recover the monophyly neither of Neogastropoda nor of Littorinimorpha, albeit these results lacked statistical support (Fig. 4). As in the phylogenetic analyses based on the 13-protein and allnuc data sets, the monophyly of each analyzed family was recovered with strong support with the exception of that of Turridae (due to the inclusion of Terebridae). Phylogenetic relationships among families were largely unresolved, and monophyly at the superfamily level could not be recovered.

### Phylogenetic relationships of Neogastropoda based on combined mt and nuclear sequences

**Figure 5:**
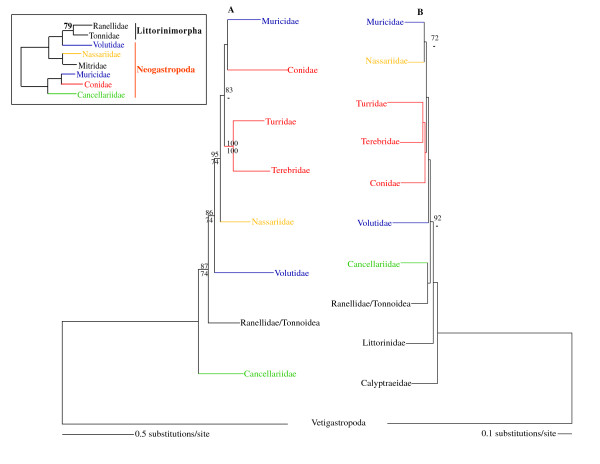
**Phylogenetic relationships within Gastropoda**. (A) ML phylogram based on the all combined data set. (B) ML phylogram based on the partial combined data set. Numbers in the nodes correspond to ML bootstrap proportions (above branches) and BI posterior probabilities (below branches). Only values above 70% are represented. The inset shows a ML topology based on fragments of mt and nuclear data (adapted from Fig. Two of Colgan *et al*., 2007).

Phylogenetic relationships within Caenogastropoda were further evaluated based on a data set (all combined) that concatenated the complete nucleotide sequences of all mt genes with partial ones of four nuclear genes (Fig. 5A). The reconstructed ML (-lnL = 102152.98) and BI (-lnL = 100152.06) trees based on the all combined data set (using different models of evolution for each partition; see Table 2) were congruent with those based on the 13-protein and all nuc data sets (Figs. 2 and 3). They only differed in the relative phylogenetic position of Conidae, (Fig. 5A versus Figs. 2 and 3). The nucleotide sequences of the genes of the four mt protein data set were also combined with partial sequences of four nuclear genes [21] into a single data set (partial combined). The reconstructed ML (-lnL = 52671.57) and BI (-lnL = 51166.67) trees were rather unresolved. Among analyzed Littorinimorpha lineages, Littorinidae and Calyptraeidae were recovered in a relative basal position, and only Ranellidae (Tonnoidea) was placed within Neogastropoda, as sister group of Cancellariidae (Fig. 5B). Conoidea was recovered as a monophyletic group, although without statistical support.

**Table 2:** Best-fit evolutionary models for each data set using PROTTEST v1.3 [71] and MODELTEST v.3.7 [72].

**Data set**	**Evolutionary model**
*amino acids*	

13-protein	MtArt I+G+F
4-protein	MtArt I+G+F
	
*nucleotides*	

13-protein (allnuc)	GTR+I+G
13-protein + 4-nuclear (allcombined)	TIM+I+G
4-protein + 4-nuclear (partialcombined)	TVM+G

*partitions allcombined*	
tRNA	TVM+G
rRNA	TVM+I+G
protein	GTR+I+G
nuclear	TrN+I+G

*partitions partial combined*	
tRNA	TVM+G
rRNA	TVM+I+G
protein	TVM+I+G
nuclear	TrN+I+G

### Testing alternative phylogenetic hypotheses

Given that phylogenetic analyses based on the most resolving (13-protein) data set failed to recover consistently the monophyly of several traditional morphological groups including Neogastropoda, Conoidea, Muricoidea, and Turridae, we further tested their validity evaluating different alternative topologies (Additional file 2). We also tested several alternative phylogenetic hypotheses on neogastropod relationships previously reported in the literature (Additional file 2; [8,9,21]). Alternative topologies were built imposing constraints on the ML topology (Fig. 2). For instance, the monophyly of Neogastropoda was imposed by forcing the tonnoidean to be positioned in a basal position with respect to the remaining Neogastropoda, and by keeping identical all remaining phylogenetic relationships as recovered in the ML tree.

The SH, AU and KH tests rejected the following alternative topologies: (1) the ML hypothesis of Colgan (topology adapted from Fig. Four of Colgan *et al*., 2007); (2) the morphology-based phylogeny of Kantor [8], and (3) monophyly of Neogastropoda, and all analyzed superfamilies/families within the group. In contrast, several alternative topologies were found to be not significantly different from the ML tree: (1) the monophyly of Conoidea; (2) the morphology-based phylogeny adapted from Ponder and Lindberg [9]; (3) the monophyly of Turridae, and (4) the monophyly of Muricoidea (Additional file 2). Finally, it is noteworthy that the monophyly of Neogastropoda was rejected under the AU and KH tests but not by the SH test (Additional file 2).

### Phylogenetic performance of mitochondrial genes

ML phylogenies were reconstructed at the nucleotide level based on each of the mt protein-coding (including all codon positions), each the two rRNA genes, and a combined data set including all mt tRNA genes. The number of congruent internal branches (i.e. nodes) between the individual phylogenies, and the whole-mt genome topology (Fig. 3) was used as a measure of the phylogenetic performance of mt genes (Table 3). None of the individual gene analysis recovered the 12 nodes (A-L) of the reference topology (Table 3 and Fig. 3). Individual genes typically recovered between 2–5, and 0–6 congruent nodes to the reference topology with bootstrap supports above/equal or below 50%, respectively. The three nodes (F, H, and L) were consistently recovered by most genes whereas others (A-D, G, I, J) were recovered only by 1–2 genes. The *cox2*, *atp6*, and *nad4 *genes recovered the highest number of congruent nodes, whereas the combined *tRNA *genes *cox1*, and *atp8 *showed the lowest phylogenetic performance.

**Table 3:** Phylogenetic performance of each mitochondrial gene.

Gene (a)	no. congruent branches BP≥50 (b)	no. congruent branches BP≤50(c)	total no. congruent branches	A	B	C	D	E	F	G	H	I	J	K	L
*cox2*	4	6	10	*			*	**	**	*	**	*	*	*	**
*atp6*	4	2	6					*	**		**		**	*	**
*nad4*	5	0	5			**		**	**		**				**
*rrnL*	3	2	5					**	**		**			*	**
*nad1*	4	0	4					*	**		**				**
*nad2*	3	1	4					**	**		**	*			**
*nad4L*	3	1	4						**		**				**
*nad5*	3	1	4					*	**		**				**
*nad6*	3	1	4					*	**		**				**
*cob*	3	1	4					*	**		**			*	**
*nad3*	3	0	3						**		**				**
*rrnS*	3	0	3						**		**				**
*cox3*	3	0	3						**		**				**
*atp8*	2	1	3	*					**		**				
*cox1*	2	0	2						**						**
tRNAs	2	0	2						**						**
	
**Reference topology**	branches BP≥50	branches BP≤50	total no. branches	A	B	C	D	E	F	G	H	I	J	K	L
													
	5	7	12	*	*	*	*	**	**	*	**	*	**	*	**

** branches with ML bootstrap proportions (BP) ≥50 in a total of 12 of the reference topology (ML tree 14,156 bp) complete mitochondrial genome)

* branches with ML BP ≤50 in a total of 12 correspondent to the reference topology

(a) Genes are ranked by the number of congruent branches with the reference topology

(b) Number of branches with BP≥50 congruent with the reference topology

(c) Number of branches with BP≤50 congruent with the reference topology

A-L Branches in the reference topology (Fig. 3)

## Discussion

### Monophyly of Neogastropoda

Recent molecular phylogenetic analyses (e.g. [28]) are challenging our view of gastropod systematics and many traditional groups (e.g. Pulmonata), widely accepted as monophyletic, are now seriously questioned [44-46]. As a result, a general perception is emerging that many morphological characters previously used to infer phylogenetic relationships among gastropod main lineages may be homoplasious [13,28], and that gastropod phylogeny needs to be revisited. For instance, our phylogenetic analyses recover Vetigastropoda as the closest living sister group to Caenogastropoda with strong quantitative support. This result challenges the traditional hypothesis that proposes Heterobranchia (i.e. the paraphyletic Heterostropha, together with Opisthobranchia and Pulmonata) as the sister group to Caenogastropoda [5,9,47,48]. The addition of nuclear data is needed to further confirm these new phylogenetic hypotheses.

Despite their remarkable present diversity, neogastropods have been traditionally recognized as a natural group because they share several key morphological features [1,5,10,11,24]. For instance, a recent phylogeny of Caenogastropoda based on 55 taxa and 164 morphological characters (particularly of external anatomy and eusperm) recovered neogastropods as a monophyletic group with maximal support [5]. In contrast, molecular phylogenetic studies have generally rendered rather unresolved caenogastropod phylogenies that challenged, albeit weakly, the widely accepted monophyly of Neogastropoda (e.g. [19,21]).

The molecular phylogenetic analyses performed in this study recovered different Littorinimorpha species (*Cymatium parthenopeum*, *Calyptraea chinensis*, and *Littorina saxatilis*) within Neogastropoda, thus questioning the monophyly of both main caenogastropod lineages. However, among these results, only the inclusion of the tonnoidean species *Cymatium parthenopeum *within Neogastropoda in the phylogeny based on the 13-protein data set received strong quantitative support (and was further confirmed by the AU and KH tests). Depending on the data set, Tonnoidea has been placed as closely related to Neogastropoda (e.g based on morphology+molecules; [5]), or within Neogastropoda in a relatively basal position either alone (based on the 13-protein and all combined data sets; this study), as sister group of Volutidae (based on partial mt and nuclear genes; [21]), or as sister group of Cancellariidae (based on the allnuc, 4-protein, and partial combined data sets; this study).

The unresolved conflict on the monophyly of Neogastropoda between morphological and molecular phylogenies suggests either that shared morphological characters of Neogastropoda are homoplasious (convergent or plesiomorphic) or that, thus far, the analyzed molecular data sets were not informative enough for resolving the phylogenetic question at hand. Regarding the possibility that morphological homoplasy is commonplace in gastropods, recent studies on the embryonic development of the valve of Leiblein (a gland that acts as an oesophageal valve binding together food particles) in Buccinidae [49] and Muricidae [12,40] demonstrated different origins of this organ in both neogastropod families. These findings indicated that this synapomorphy of Neogastropoda could be uncertain [50], and prompt for the re-evaluation of other synapomorphies defining Neogastropoda.

Regarding the possibility that molecular data sets need to be improved, the phylogenetic analyses performed here show that the effort in increasing the number of analyzed positions with respect to previous studies was not enough to achieve conclusive results regarding phylogenetic relationships within Neogastropoda. Internal branches connecting main caenogastropod/neogastropod lineages were relatively short. Such phylogenetic pattern is in agreement with the fossil record, which supports a rapid radiation at the origin of Neogastropoda [11,24]. Therefore, it would be expected low resolution at this part of the caenogastropod tree regardless of the amount of sampled characters (but observe the increase in resolution in the ML tree based on 13-protein data set with respect to that based on the 4-protein data set, and to the individual phylogenetic analyses – see below). It is also noteworthy that internal branches in the phylogenetic tree based on the 13-protein data set (Fig. 2) presented higher bootstrap support than the ones in the phylogenetic tree based on the allnuc data set (Fig. 3). This result is in agreement with the preferential use of amino acids to reconstruct deep phylogenies because they (1) have a larger character-state space compare to nucleotides, (2) show a slower rate of evolution compare to silent substitutions, and (3) are less influenced by compositional bias [51]; [26]; [52]. Our phylogenetic analyses show that addressing the question on the monophyly of Neogastropoda is tightly connected with resolving phylogenetic relationships among Caenogastropoda main lineages, and hence that future phylogenetic studies based on complete mitochondrial genome sequence data will require a thorough representation of all Caenogastropoda main lineages.

### Phylogenetic relationships within Neogastropoda

All phylogenetic analyses agreed on the monophyly of the different analyzed neogastropod families except Turridae (due to the inclusion of Terebridae). A recent molecular study [53] based on partial mitochondrial and nuclear genes, and a rather thorough sampling of both families also supported this view. At higher taxonomic levels, the phylogenetic analysis of the different data sets rendered (1) rather unresolved topologies; (2) showed markedly different phylogenetic relationships based on each data set, and (3) failed to recover the monophyly of neogastropod superfamilies. Only the phylogeny based on the 13-protein data achieved some degree of resolution regarding superfamily phylogenetic relationships. According to this phylogeny, Cancellarioidea occupies a basal position with respect to remaining neogastropods. This basal position was already suggested in some previous morphological studies [8,54]. In particular, it has been shown that the gland of Leiblein is separated from the oesophagus in all neogastropods but Cancellarioidea, which retains the primitive condition [8,12]. Other phylogenetic studies recovered Buccinoidea or Nassariidae+Mitridae as the most basal neogastropod superfamily based on morphology or combined morphological + molecular data sets, respectively [5]. A phylogeny based on partial mt and nuclear genes [21] recovered a basal politomy at this level of the caenogastropod tree, and leaved the controversy unsettled since either Volutidae (as sister group of Tonnoidea) or Cancellariidae (as sister group of Calyptraeidae) could be the most basal neogastropod lineages.

Both, ML and BI trees based on the 13-protein data set suggest that the monophyly of the superfamily Muricoidea is doubtful because Muricidae and Volutidae are not recovered together. All performed phylogenetic analyses based on other data sets also failed to recover Muricoidea as a monophyletic group. This result was already suggested in several previous studies [5,15,21]. The status of the superfamily Conoidea remains uncertain. The BI tree based on the 13-protein data set (not shown), as well as ML and BI trees based on the allnuc data set recovered the families Turridae, Terebridae and Conidae together, albeit with no strong statistical support. ML based on the 13-protein data set fail to recover the monophyly of Conoidea but this hypothesis could not be confidently rejected by statistical tests. Overall, our results indicate that neogastropod relationships are far from being settled (in "a state of flux" as described by [5]).

### Phylogenetic performance of mitochondrial genes

The relative merit of the different genes in recovering a phylogeny is associated with their respective substitution rates, and thus the taxonomic level at which higher resolution is achieved. Several studies evaluated the phylogenetic performance of mt genes in recovering vertebrate phylogeny [25-27,55]. According to these studies, *nad4 *and *nad5 *were ranked generally as the best performing mt genes, whereas *atp8*, *nad6*, and *nad4L *genes revealed the lowest phylogenetic performance. In this study, we evaluated the phylogenetic performance of individual mt genes in recovering caenogastropod phylogeny, and found some remarkable differences when compared with results from the above-mentioned studies in vertebrates. The gene *cox2 *showed the largest number of congruent branches with the whole-genome reference topology, whereas it was never recovered as having the best phylogenetic performance in vertebrates [25-27,55]. In our study, the mt tRNA genes showed the lowest performance, whereas in Mueller [27] these genes demonstrated a medium ability to recover the reference topology. The mt gene *cox1 *also showed a very low performance in recovering caenogastropod phylogeny, whereas it was ranked as a good gene for recovering the expected vertebrate tree. All studies agreed in pointing mt *atp8 *as having low phylogenetic performance. In any case, combining all mt genes into a single data set always rendered the most resolved trees, supporting the use of complete mt genome data for addressing caenogatropod phylogeny.

### Evolution of mitochondrial gene order of Neogastropoda

Shared mt gene orders may be useful for phylogenetic inference particularly at higher taxonomic levels [56]. Gastropods exhibit an important degree of variation in mt gene organization compared with other animals [28,57-59]. Main events of gene rearrangement occurred at the origin of Patellogastropoda and Heterobranchia, whereas fewer changes occurred between cephalopods (here represented by *Octopus*) and the ancestors of Vetigastropoda (only tRNAs D, C and N) and Caenogastropoda (a large single inversion, and translocations of the tRNAs D and N). Within Heterobranchia, gene order seems to be relatively conserved and gene rearrangements are mostly related with transposition of tRNA genes [28]. All eight genomes sequenced in this study exhibit an unusually conserved gene order only contradicted by two unrelated tRNA translocations found in *Fusiturris similis *and *Terebra dimidiata *(Fig. 1). Given the high rates of gene rearrangement among gastropod main lineages, the conserved gene order found between all neogastropod and Littorinimorpha mt genomes suggests a shared evolutionary history between the analyzed lineages.

## Conclusion

As more molecular data are analyzed, the monophyly and phylogenetic relationships of Neogastropoda are emerging as rather elusive evolutionary questions to be resolved. This is likely due to a rapid origin of Neogastropoda lineages back into the Early Cretaceous. Our phylogenetic analyses based on mt genome sequence data confirm previous molecular studies that contradicted the monophyly of Neogastropoda. This group used to be considered well-supported based on several morphological synapomorphies [5,8,10,11,60]. However, recent studies question the validity of some of these synapomorphies suggesting that Neogastropoda could be an assembly of taxa erroneously grouped on the basis of convergent morphological characters [50]. If confirmed, molecular data may become particularly useful for determining the Caenogastropoda phylogeny. In this regard, future studies testing the monophyly of Neogastropoda based on mt genome sequence data will need to include a thorough sampling of main caenogastropod lineages. Our ML and BI analyses based on the 13-protein data set, produced topologies better resolved than previous studies using fragments of nuclear and mt sequence data. Moreover, results of phylogenetic performance indicated that none of the individual mt genes recovered the whole-genome reference ML tree, which may indicate that larger data sets may provide more informative sites for this particular phylogenetic question. However, in parallel to the steady accumulation of new mt genome data, new nuclear data (e.g., Expressed Sequence Tags – ESTs) need also to be gathered, and only the combination of both types of sequence data might render better results.

## Methods

### Taxon sampling

To assess phylogenetic relationships within Neogastropoda, the entire mt genome was sequenced in the following species representing four out of the six currently recognized neogastropod superfamilies. *Bolinus brandaris *(L., 1758) and *Cymbium olla *(L., 1758) both belonging to the superfamily Muricoidea; *Nassarius reticulatus *(L., 1758) (superfamily Buccinoidea); *Cancellaria cancellata *(L., 1767) (superfamily Cancellarioidea), and *Fusiturris similis *(Bivona, 1838), *Conus borgesi *[61], and *Terebra dimidiata *(L., 1758), all belonging to superfamily Conoidea.

In order to test the monophyly of Neogastropoda, the mt genomes of two species belonging to the closely related order Littorinimorpha (Caenogastropoda) were also sequenced: *Cymatium parthenopeum *(von Salis, 1793) (superfamily Tonnoidea) and *Calyptraea chinensis *(L., 1758) (superfamily Calyptraeoidea). The mt genome of the former species was sequenced completely, whereas only about 8,500 bp were sequenced from the mtDNA of *Calyptraea chinensis *due to unsuccessful PCR amplification of the remaining portion of the genome. A similar case of partial mt genome sequencing due to failed PCR amplification was reported for *Littorina saxatilis *[43]).

In addition, we included in the phylogenetic analyses the following gastropod entire mt genomes that are available in GenBank: (1) superfamily Conoidea:*Conus textile *(NC_009797, [35] and *Lophiotoma cerithiformis *(NC_008098, [36]); (2) superfamily Buccinoidea: *Ilyanassa obsoleta *(NC_007781, [37]); (3) superfamily Muricoidea: *Thais clavigera *(NC_010090[38]), and *Rapana venosa *(NC_011193[39]), (4) Opisthobranchia: *Aplysia californica *(NC_005827, [62]) and *Roboastra europaea *(NC_004321, [57]); (5) Pulmonata: *Albinaria coerulea *(NC_001761, [63]); (6) Vetigastropoda: *Haliotis rubra *(NC_005940, [64]), and (7) Patellogastropoda: *Lottia digitalis *(NC_007782, [37]). A partial fragment of *Littorina saxatilis *(Litorinimorpha: Littorinoidea; AJ_132137, [43]) mt genome available in GenBank was also included in some phylogenetic analyses.

### PCR amplification and sequencing

Total DNA was extracted using either a standard phenol-chloroform DNA extraction protocol with cetyltrimethylammonium bromide (CTAB) [65], or commercial extraction kits (DNA Easy extraction Kit, Qiagen; ChargeSwitch gDNA Micro Tissue Kit, Invitrogen). Several pairs of primers (see Additional file 3) were used to amplify by PCR, contiguous and overlapping fragments that covered the entire mt genomes. PCR amplifications were carried out with the PCR Extender System (5 Prime) in 25 μl reactions containing 10× PCR Extender buffer, 0.2 mM of each dNTP, 2 mM of MgCl_2_, 0.2 μM of each primer, 1–2 μl of template DNA, and Taq DNA polymerase (1 unit). The following general profile was used: an initial denaturing step at 94°C for 2 min; 35 to 40 cycles of denaturing at 94°C for 20 s, annealing at 42–55°C for 20 s, and extending at 68°C for 60 s per kb; and a final extending step at 68°C for 7 min.

PCR amplicons were purified by ethanol precipitation. Those fragments with sizes < 2500 bp were either directly sequenced with the corresponding PCR primers or cloned into pGEM-T Easy Vector System (Promega), and sequenced with modified universal M13 primers (see Additional file 3). Longer PCR products were directly sequenced using a primer walking strategy. Sequencing was performed in an automated sequencer (ABI PRISM 3700) using the BigDye^® ^Terminator v3.1 Cycle Sequencing Kit (Applied Biosystems), and following manufacturer's instructions. The obtained sequences averaged 900 bp in length, and each sequence overlapped the next contig by about 150 bp. In no case were differences observed between overlapping regions.

### Phylogenetic analyses

Gene annotation was performed by sequence comparison with other mt genomes of gastropods. Open reading frames helped to delimit start and stop codons of the protein coding genes. Cloverleaf secondary structures of all tRNAs were reconstructed either by hand or with TRNASCAN-SE 1.21 [66]. Ribosomal RNA limits were based on the boundaries of the flanking genes. The new sequences reported in this paper have been deposited at GenBank under accession numbers EU827194 (*Bolinus brandaris*), EU827195 (*Cancellaria cancellata*), EU827199 (*Cymbium olla*), EU827197 (*Fusiturris similis*), EU827201 (*Nassarius reticulatus*), EU827196 (*Terebra dimidiata*), EU827198 (*Conus borgesi*), EU827200 (*Cymatium parthenopeum*), and EU827193 (*Calyptraea chinensis*).

Separate alignments of the nucleotide and deduced amino acid sequences of each mt protein-coding gene were constructed with CLUSTAL X version 1.83 using default parameters [67], and verified by eye in order to maximize positional homology. Alignment ambiguities were excluded from phylogenetic analyses using GBLOCKS version 0.91b [68,69]. Five different data sets (available from the authors upon request) were analyzed: **(1) 13-protein data set**: the deduced amino acid sequences of the 13 protein-coding mt genes of the 12 available Neogastropoda, two Opisthobranchia (*Aplysia californica *and *Roboastra europaea*), one Pulmonata (*Albinaria coerulea*), one Vetigastropoda (*Haliotis rubra*). and one Patellogastropoda (*Lottia digitalis*) complete mt genomes were concatenated into a single data set. The alignment consisted of 3,170 positions, of which 935 were constant, and 1,542 were parsimony informative; **(2) allnuc data set**: the nucleotide sequences of the rRNA, tRNA and protein-coding genes of all available complete mt genomes of neogastropods, one Ranellidae, and one Vetigastropoda, were concatenated into a single data set of 14,156 positions. Of these, 5,371 positions were constant, and 6,407 were parsimony informative; **(3) 4-protein data set**: the available partial mitochondrial genome sequences of two caenogastropod taxa, *Littorina saxatilis *(AJ_132137[43]) and *Calyptraea chinensis *(this study) only have in common four protein-coding mitochondrial genes (*cox1*; *cox2*; *atp6*, and *atp8*). The deduced amino acid sequences of these four genes from these two taxa together with those of the taxa included in the 13-protein data set were concatenated into a single data set, which produced an alignment of 982 positions. Of these, 366 were constant, and 408 were parsimony informative; **(4) allcombined data set**: the nucleotide sequences of the rRNA, tRNA and protein-coding genes of complete mt genomes of seven neogastropods (Muricidae, Cancellariidae, Volutidae, Turridae, Nassariidae, Terebridae, and Conidae), one Ranellidae, and one Vetigastropoda, all from this study, combined with partial sequences of the nuclear genes *28S rRNA*, *18S rRNA*, *histone H3*, and *elongation factor 1α *(nuclear sequences retrieved from GenBank from the study of Colgan *et al*, 2007) were concatenated into a single data set of 16,937 positions. Of these, 7,996 were constant and 5,550 were parsimony informative; **(5) partial combined data se**t: the nucleotide sequences of 4 protein-coding genes (*cox1*; *cox2*; *atp6*, and *atp8*) of seven neogastropods (Muricidae, Cancellariidae, Volutidae, Turridae, Nassariidae, Terebridae, and Conidae), one Ranellidae, and one Vetigastropoda, all from this study, combined with partial sequences of the nuclear genes *28S rRNA*, *18S rRNA*, *histone H3*, and *elongation factor 1α *(nuclear sequences retrieved from GenBank from the study of Colgan *et al*., 2007) were concatenated into a single data set of 8,462 positions. Of these, 4,113 are constant and 2,523 were parsimony-informative.

The best-fit models of evolution for the amino acid and nucleotide sequence data sets were selected using the Akaike Information Criterion (AIC) [70] with PROTTEST v1.3 [71] and MODELTEST v.3.7 [72], respectively (see Table 2). ML trees were inferred based on the different data sets with PHYML v3.0 [73]. The robustness of the inferred trees was tested using non-parametric bootstrapping (BP) of 500 pseudoreplicates.

BI trees were inferred based on the different data sets with MRBAYEs v3.1.2 [74]. Four Metropolis-coupled Markov chain Monte Carlo (MCMC) analyses were run for 1 million generations, and sampled every 100 generations. The mixed model option was used for the amino acid sequence data sets. For the analysis of nucleotide sequence data sets, all 13 protein-coding genes, ribosomal RNAs, and tRNAs were considered as three different partitions in the allnuc data set. A fourth partition of nuclear genes was considered in the combined data sets. The option (preset = variable) that allows rates to differ across partitions was used with the "unlink" command. Burn-in included the first 100,000 generations for the 13-protein, all combined, and partial combined data sets, whereas the first 50,000 generations were discarded for the 4-protein, and allnuc data sets. Statistical support was assessed using Bayesian posterior probabilities (BPPs).

In order to evaluate alternative hypotheses on neogastropod phylogenetic relationships found in the literature, the approximately unbiased (AU) [75], the Kishino-Hasegawa (KH) [76], and the Shimodaira-Hasegawa (SH) [77] tests were performed based on the 13-protein data set. These tests are implemented in CONSEL v0.1i [78] and use log-likelihoods of site-patterns of the trees estimated with PAML v.4 [79].

### Phylogenetic performance of mt genes

In order to determine whether the allnuc data set had better phylogenetic performance than individual mt genes, ML analyses were performed with PHYML v2.4.4 [73]. Individual analyses were based on each of the 13 individual mt protein-coding genes, each of the two mt ribosomal genes, and mt tRNAs (all mt tRNAs were concatenated into a single data set). The best-fit models and parameters for each of these data sets were estimated under the AIC [70] as implemented in MODELTEST v.3.7 [72]. The robustness of the inferred trees was tested using BPs of 500 pseudoreplicates. Following Mueller [27], we compared the number of resolved nodes in each of the 50% bootstrap consensus ML trees that were inferred based on individual gene analyses to the number of resolved nodes in the 50% bootstrap consensus ML topology (Fig. 3) that was reconstructed based on the allnuc data set, as a measure of phylogenetic performance of the different individual data sets.

## Authors' contributions

RLC organized the sampling, and carried out the molecular work. CG conducted gene annotation and analyzed gene rearrangements. RLC performed phylogenetic reconstructions. RZ designed and developed the study. RLC, CG, and RZ wrote the manuscript. All authors read and approved the final version of the manuscript.

## Supplementary Material

Additional file 1 **Table S1**. Neogastropoda taxonomic classification. Click here for file

Additional file 2 **Table S2**. Testing alternative topologies. Click here for file

Additional file 3 **Appendix**. List of primers used in this study. Click here for file
