# Supplementary material for: Neogastropod phylogenetic relationships based on entire mitochondrial genomes
Source: BMC Evol Biol. 2009 Aug 23;9:210. doi: 10.1186/1471-2148-9-210 (PMC2741453; doi:10.1186/1471-2148-9-210)
Supplement: Additional file 1 — Table S1. Neogastropoda taxonomic classification. [file 1471-2148-9-210-S1.pdf]

	Thiele, 1929-35/ Wenz, 1938-44	Ponder, 1973		Kantor, 1996		Bouchet & Rocroi, 2005	
**Superfamily**	**Family**	**Superfamily**	**Family**	**Superfamily**	**Family**	**Superfamily**	**Family**
**MURICACEA**	Muricidae	**MURICACEA**	Muricidae=Thaididae	**MURICOIDEI**	Muricidae	**MURICOIDEA**	Muricidae
	Coralliophilidae		Magilidae = Coralliophilidae, Rapidae		Coralliophilidae		Babyloniidae
**BUCCINACEA**	Columbellidae		Pyrenidae = Columbellidae		Columbellidae		
	Buccinidae		Buccinidae		Buccinidae		
	Melongenidae		Galeolidae = Melongenidae, Volemidae				
	Nassariidae		Nassariidae		Nassariidae		
	Fasciolariidae		Fasciolariidae		Fasciolariidae		
			Vasidae				
**VOLUTACEA**	Mitridae		Mitridae		Mitridae		Mitridae
	Turbinellidae		Turbinellidae = Vasidae, =Xancidae		Turbinellidae		Turbinellidae
	Harpidae		Harpidae		Harpidae		Harpidae
	Volutidae		Volutidae		Volutidae		Volutidae
			Colubrariidae= Fusidae		Colubrariidae		
	Marginellidae		Marginellidae				Marginellidae
					Costellariidae		Costellariidae
					Pleyoptygmatidae		Pleyoptygmatidae
					Melapiidae		
							Cysticidae
							Strepsiduridae
			Volutomitridae				Volutomitridae
	Olividae		Olividae (**subfamilies**: Olivinae; Ancilinae; Olivellinae; Pseudolivinae)	**OLIVELLIDOIDEI**	Olividae	**OLIVOIDEA**	Olividae
					Olivellidae		Olivellidae
			Columbariidae			**BUCCINOIDEA**	Columbellidae
							Buccinidae
			Vexillidae				Melongenidae
							Nassariidae
							Fasciolariidae
							Colubrariidae
	Cancellariidae	**CANCELLARIACEA**	Cancellariidae	**CANCELLARIOIDEI**	Cancellariidae	**CANCELLARIOIDEA**	Cancellariidae
				**PSEUDOLIVIDOIDEI**	Pseudolividae	**PSEUDOLIVOIDEIA**	Pseudolividae
**CONACEA**	Conidae	**CONACEA**	Conidae	**CONOIDEI**	Conidae	**CONOIDEA**	Conidae
	Terebridae		Terebridae		Terebridae		Terebridae
			Turridae		Turridae		Turridae
					Pseudomelatomidae		Pseudomelatomidae
					Strictispiridae		Strictispiridae
					Clavidae		Clavatulidae
							Drilliidae
