# Supplementary material for: Neogastropod phylogenetic relationships based on entire mitochondrial genomes
Source: BMC Evol Biol. 2009 Aug 23;9:210. doi: 10.1186/1471-2148-9-210 (PMC2741453; doi:10.1186/1471-2148-9-210)
Supplement: Additional file 2 — Table S2. Testing alternative topologies. [file 1471-2148-9-210-S2.pdf]

**Alternative topologies**	**Phylogenetic hypotheses**		13-gene data set			
			-logL	AU	KH	SH
(Outg,(Veti,(Canc,(Rane,(Volu,(Coni,((Muri,(Muri,Muri)),((Turr,(Tere,Turr)),Nass)))))));	Our ML topology based on complete mt genomes	#1	46914.17	**0.877**	**0.701**	**0.996**
(Outg,(Veti,(Canc,(Rane,(Volu,((Muri,(Muri,Muri)),(((Turr,(Tere,Turr)),Coni),Nass))))));	Conoidea monophyletic	#2	46921.54	**0.439**	**0.299**	**0.825**
(Outg,(Veti,(Rane,(Canc,(Volu,(((Muri,(Muri,Muri)),((Turr,(Tere,Turr)),Coni)),Nass))))));	Morphology-based phylogeny (adapted from Fig.5 of Ponder&Lindberg, 1997)	#7	46935.85	**0.155**	**0.134**	**0.478**
(Outg,(Veti,(Canc,(Rane,(Volu,(Coni,((Muri,(Muri,Muri)),((Tere,(Turr,Turr)),Nass)))))));	Turridae monophyletic	#3	46932.82	**0.077**	**0.058**	**0.511**
(Outg,(Veti,(Canc,(Rane,(Coni,(((Muri,(Muri,Muri)),Volu),((Turr,(Tere,Turr)),Nass))))));	Muricoidea monophyletic	#4	46933.31	**0.075**	**0.055**	**0.505**
(Outg,(Veti,(Rane,(Canc,(Volu,(Coni,((Muri,(Muri,Muri)),((Turr,(Tere,Turr)),Nass)))))));	Neogastropoda monophyletic	#5	46935.17	0.01	0.009	**0.462**
(Outg,(Veti,((Canc,((Muri,(Muri,Muri)),(Coni,(Turr,(Tere,Turr))))),((Rane,Volu),Nass))));	ML phylogenetic hypothesis (adapted from Fig. 2 of Colgan, 2007)	#8	46986.46	0.002	0.007	0.021
(Outg,(Veti,(Rane,(Canc,(((Muri,(Muri,Muri)),Volu),(((Tere,(Turr,Turr)),Coni),Nass))))));	All groups within Caenogastropoda monophyletic	#6	46976.17	<0.001	0.003	0.016
(Outg,(Veti,(Rane,(Canc,(((Coni,(Turr,Turr)),Tere),(Volu,((Muri,(Muri,Muri)),Nass)))))));	Morphology-based phylogeny Kantor, 1996	#9	47035.14	<0.001	<0.001	<0.001

Veti: Vetigastropoda; Canc: Cancellariidae; Rane: Ranellidae; Volu: Volutidae; Coni: Conidae; Turr: Turridae; Tere: Terebridae;
Muri: Muricidae;Nass: Nassariidae; Outg: outgroup
